# Differences in Characteristics and Treatment Received among Depressed Adolescent Psychiatric Outpatients with and without Co-Occuring Alcohol Misuse: A 1-Year Follow-Up Study

**DOI:** 10.1155/2011/140868

**Published:** 2011-05-23

**Authors:** Tiia Pirkola, Mirjami Pelkonen, Linnea Karlsson, Olli Kiviruusu, Thea Strandholm, Virpi Tuisku, Titta Ruuttu, Mauri Marttunen

**Affiliations:** ^1^Department of Mental Health and Substance Abuse Services, National Institute for Health and Welfare, P.O. Box 30, 00271 Helsinki, Finland; ^2^Department of Child Psychiatry, The Turku University Central Hospital, P.O. Box 52 Kiinamyllynkatu 4-8, Rak 10, 20521 Turku, Finland; ^3^Department of Adolescent Psychiatry, Helsinki University Central Hospital, P.O. Box 590, 00029 HUS, Helsinki, Finland; ^4^Department of Psychiatry, University of Helsinki, P.O. Box 590, 00029 HUS, Helsinki, Finland

## Abstract

*Objectives*. We aimed at examining the differences between depressed psychiatric adolescent outpatients with and without cooccurring alcohol misuse in psychosocial background, clinical characteristics, and treatment received during one-year followup. Furthermore, we investigated factors related to nonattendance at treatment. *Materials and Methods*. Consecutive 156 adolescent (13–19 years) psychiatric outpatients with a unipolar depressive disorder at baseline were interviewed using structured measures at baseline and at 12 months. Alcohol misuse was defined as having an AUDIT score of 8 or more points. The outpatients received “treatment as usual” of clinically defined duration. *Results*. Among depressive outpatients, poor parental support, parental alcohol use and decreased attendance at treatment associated with alcohol misuse. The severity of alcohol use as measured by AUDIT-score was the strongest factor independently predicting nonattendance at treatment in multivariate analysis. *Conclusions*. Alcohol misuse indicates family problems, has a deleterious effect on treatment attendance, and should be taken into account when managing treatment for depressive adolescent outpatients.

## 1. Introduction

Depressive disorders are common among adolescents with an estimated point prevalence of about 3% to 8%, and with a lifetime prevalence of approximately 20% by the end of adolescence [1–3]. Among adolescents, excessive substance use often co-occurs with depressive illness [4], alcohol being the most common substance used among Finnish adolescent population [5, 6]. Studies have found positive associations of alcohol use frequency and recurrent intoxication among depressed adolescents and of early-onset depressive disorders with elevated levels of later addictive substance use [5]. It has been reported that 10–25% of depressed subjects have cooccurring substance use disorder (SUD) both in general and clinical populations with clearly higher figures in clinical populations [7, 8]. 

There is evidence that comorbid conditions increase utilization of psychiatric services, complicate treatment, and have a negative impact on the course and prognosis of both disorders [9–13]. Depression and SUD each, and their cooccurrence in particular, are associated with psychosocial impairment, more severe symptoms, and increased suicidality [4, 14–19]. The growing literature emphasizes the clinical importance of substance use not reaching the diagnostic threshold in young people [10, 20] as it seems to associate with a greater number of other psychiatric symptoms [20].

Despite recognized risks of adolescent substance use among depressed juvenile patients, studies focusing on treatment of cooccurring depression and SUD are still scarce [21]. The outcome of treating either disorder alone is known to be unsatisfactory [22, 23]. Cornelius et al. [24] suggested that psychological intervention should be considered first-line treatment for this population, with pharmacotherapy offered to those who do not respond to this intervention alone. In the study by Riggs et al. [25], the combination of fluoxetine and cognitive behavioural therapy (CBT) showed superior efficacy to that of placebo and CBT for MDD according to changes of the Childhood Depression Rating Scale-Revised in adolescents with SUD. The relative gap of our knowledge is partly due to historical separation of and lack of coordination between substance and mental health treatment programs in many countries. Furthermore, in controlled treatment studies, patients with comorbid disorders are usually excluded [21, 26]. In psychiatric clinical practise, it seems that substance-related problems are not detected at all or diagnosed too late leading to a lack or delay of treatment, as Couwenbergh et al. have pointed out [27]. For the development of specific treatments for these adolescents with comorbid disorders, more research is needed.

The present study seeks to add to the limited research on treatment of depression in the context of cooccurring alcohol use and on factors related to nonattendance at treatment in depressed adolescents. We analyzed data from a Finnish naturalistic one-year follow-up study of depressed adolescent outpatients. Our aim was to examine the differences in psychosocial background characteristics and treatment received during the one-year follow-up time of depressed psychiatric outpatient adolescents with and without cooccurring alcohol misuse and to investigate factors related to nonattendance at treatment in this population.

## 2. Materials and Methods

### 2.1. Subjects and Measures

This study forms part of the Adolescent Depression Study (ADS), a collaboration study between the Department of Adolescent Psychiatry of Peijas Medical Health Care District (PMCD) of Helsinki University Central Hospital and the Department of Mental Health and Substance Abuse Services of the National Institute for Health and Welfare (former National Public Health Institute), Finland. The study protocol was accepted by the ethics committees of Helsinki University Central Hospital and PMCD.

The ADS is a naturalistic clinical research and development project on adolescent depressive mood disorders in a sample of consecutive adolescent psychiatric outpatients. The study population was drawn from the PMCD's two adolescent psychiatric outpatient clinics between February 1, 1998 and December 31, 2001. PMCD covers approximately 210,000 inhabitants (about 15% adolescents) of the Helsinki metropolitan area in southern Finland. The outpatient clinics in the PMCD offer secondary care to all 13- to 19-year-old citizens. Patients are mainly referred from schools, health care centers, and social and family counseling services. Subjects believed to have a predominant and severe substance use disorder (SUD) and the most severe eating disorders are offered treatment elsewhere in specialized units. Finland has universal access to health care, and adolescent psychiatric care is free of charge for the patient. The outpatient clinics offer eclectic psychiatric treatment including individual supportive therapy, family consultations, and psychotropic medication when appropriate. 

The screening and diagnostic procedure and the study population (*n* = 218) of the ADS study have been described in more detail previously [3, 28]. In brief, of the 774 consecutive outpatients, 660 (85.3%) were considered eligible. The exclusion criteria were age below 13 or over 19 years, mental retardation, insufficient knowledge of the Finnish language, or admission including no individual appointments. The patient sampling for ADS involved screening all patients by the Beck Depression Inventory-21 [29] and the General Health Questionnaire-36 self-report measures [30]. Studies have supported the reliability and validity of BDI-21 and GHQ-36 also among adolescent populations [31–34]. The sum scores ≥10 on the BDI-21 and ≥5 on the GHQ-36 were considered screening positives and were invited to participate. Screening positives were fully informed of the study project, and written informed consent was requested from both participants and their parents from those under 18 years of age. Of the eligible patients, 373 (56.5%) were screen positives. Of the screen positives, 221 (59.2%) agreed to participate in the study and were then interviewed. Almost all of the interviewed subjects (*N* = 218) had an ongoing episode of either unipolar or bipolar depression at baseline evaluation and were recruited to the study. Adolescent who declined to participate were similar to the study subjects in terms of age, sex, and parental socioeconomic status while they tended to have lower BDI-21 (19.0 versus 21.0, *z* = −1.93,   *df* = 371,   *P* = .05) and lower GHQ-36 (21.0 versus 24.0, *z* = −1.98,   *df* = 367,   *P* = .05) median sum scores [3, 8].

Data were obtained by interviewing the adolescents themselves and collecting additional background data from the clinical records. In clinical practice, at baseline, parents were offered at least one consultation appointment and data on adolescent's as well as parental problems were collected. Special efforts were made in order to confirm that all data in clinical records were appropriate, right, and timed. In a naturalistic manner, after the comprehensive baseline evaluation (T1), the outpatients received “treatment as usual” of clinically defined duration. The study subjects were reevaluated in 6 months and one year (T2). The median time interval between T1 and T2 was 59.5 weeks (interquartile range (IQR), 57.0–63.0 weeks). 

Excluded for the analyses of this study were those subjects (1) who were diagnosed either at the baseline or at later diagnostic interviews as having bipolar disorder (*n* = 21), (2) with missing data of Alcohol Use Disorder Identification Test (AUDIT) [35] at baseline (*n* = 12), and (3) who did not participate in the one-year interview (*n* = 29). Consequently, the final study population of this study comprised of 156 patients with diagnosed unipolar depression. For the analyses, the subjects were classified into two groups according to level of self-reported alcohol use at baseline: (1) nonmisusers (*n* = 86) had AUDIT score of less than 8 and (2) alcohol misusers (*n* = 70) had AUDIT score of 8 or more. The cutoff point of 8 in AUDIT was chosen based on previous research [35, 36]. AUDIT is a self-report measure to assess alcohol-related problems, which is a commonly used and a clinically meaningful instrument in ordinary clinical practice. The AUDIT has reasonable psychometric properties among adolescents [35, 37–39].

### 2.2. Sociodemographic, Diagnostic, and Clinical Characteristics at Baseline

Sex and age at baseline were taken directly from the data. The socioeconomic status (SES) of the adolescent's parents was classified as follows: upper middle class, lower middle class, working class, or other (including students, unemployed, retired (pensioner), others not defined) [40]. Parents' divorce, alcohol use, or mental health problems were recorded based on the information received from the subjects and/or the parents. Social support was assessed by the Perceived Social Support Scale-Revised (PSSS-R) [41]. PSSS-R measures persons' subjective perceptions of social support and emotional closeness, not actual number of supportive contacts. It has been shown to be a useful method in assessing perceived social support in Finnish adolescents [42, 43].

The Schedule for Affective Disorders and Schizophrenia for School-Aged Children—Present and Life-time version (K-SADS-PL) [44] was used to assess present and lifetime episodes of DSM-IV Axis I disorders. The DSM-IV Axis II disorders were assessed with the Structural Clinical Interview for DSM-IV Axis II Disorders (SCID-II) interview [45]. For the analyses, Axis II diagnoses were dichotomized (yes/no Axis II diagnose). Axis III diagnoses were dichotomized according to whether the patient had any doctor-diagnosed medical condition or not. Nine researchers, who were expert level clinicians (educated psychiatrists and psychologists), conducted the diagnostic interviews, and all the research diagnoses were confirmed in a subsequent diagnostic meeting. Interrater reliability, assessed using 15 randomly selected videotaped interviews, was good for mood disorder diagnoses (weighted kappa [46, 47] for MDD, other mood disorder, no mood disorder 0.87 (95% CI 0.81, 0.93)) [3]. 

Current psychosocial functioning (Global Assessment of Functioning, GAF) was assessed according to the DSM-IV Axis V definitions [48], that is, indicating the level of functioning at the time of the interview. For the group comparisons, the GAF score was used as a dichotomous variable, with a cut-point of 60 indicating “at least moderate impairment” [3, 8].

Severity of depression was measured by Beck Depression Inventory (BDI-21) [29] total sum-scores (range 0 to 60). Alcohol misuse was assessed by the AUDIT sum-score (range 0 to 40), severity of anxiety symptoms was measured by using the sum-score (range 0 to 63) of the Beck Anxiety Inventory (BAI) [49] and severity of suicidality by using the sum-score of the Scale for Suicidal Ideation (SSI) [50].

Age of onset of depression was recorded based on the information collected from the clinical records and at the interview.

### 2.3. Treatment

As the study was naturalistic, the outpatients received “treatment as usual” of clinically defined duration in a general adolescent psychiatric setting of Finnish secondary health care. The treatment team consists of a psychiatrist specialized in adolescent psychiatry, a psychologist, one or more psychiatric nurses, and a social worker. The treatment modalities used at the outpatient clinics consisted of individual supportive therapy, family consultations, and psychotropic medication when appropriate. The treatment always begun with an evaluation phase. The information of treatment received was gathered from the one-year follow-up data. At the one-year follow-up time, 65,4% of the subjects were continuing their treatment. 

#### 2.3.1. Psychosocial Treatment

Following data were gathered from the medical records: number of scheduled and kept individual appointments, number of scheduled and kept family/network appointments, *intensity *(kept individual appointments/month), and *attendance at treatment* (proportion of kept to scheduled individual appointments).

#### 2.3.2. Pharmacological Treatment

The information of pharmacological treatment was used as a dichotomous variable (yes/no prescribed medication during the treatment/one-year follow-up time) in the following medication groups: (1) Serotonin reuptake inhibitors (SSRI:s), (2) other antidepressant medication, (3) anxiolytics, (4) antipsychotics, and (5) other psychotropic medication.

*Combined treatment *was classified as follows: (1) individual psychotherapy, (2) individual psychotherapy and family counseling, (3) individual psychotherapy and medication, and (4) individual psychotherapy, family counseling, and medication. The information of *hospitalization* was used as a dichotomous variable (yes/no hospitalization during the one-year follow-up time). In addition, the number of hospitalization days during the treatment was recorded.

### 2.4. Diagnostic and Clinical Characteristics at One-Year Followup

At one-year followup, the severity of depression was measured by the BDI-21. Total sum score and the level of psychosocial functioning (Global Assessment of Functioning, GAF) were assessed according to the DSM-IV Axis V definitions. Level of alcohol use was assessed by using the AUDIT sum-score. The diagnostic status of the depressive disorder at the time of the one-year follow-up diagnostic interviews was rated as follows: (1) recovery, (2) persistent depression, and (3) recurrence during the study period. Two months of 1 or no symptoms (no depressed or irritable mood or anhedonia) was defined as recovery. Lack of recovery during the one-year follow-up period was defined as persistent depression. Recurrence was defined as a new depressive episode emerging after the beginning of recovery.

### 2.5. Statistical Analysis

To analyze differences between alcohol misusers and nonmisusers, we used Chi-square for the categorical variables, *t*-test for normally, and Mann-Whitney *U* tests for non-normally distributed continuous variables. To analyze factors independently associating with treatment attendance, a logistic regression model was conducted with response variable (=attendance) equal to the binomial proportion of kept to scheduled individual appointments. Possibly compliance-related clinical and treatment factors were the explanatory variables including AUDIT and BDI scores at baseline, perceived social support from the family, psychosocial functioning (GAF score), comorbid axis I and axis II diagnoses at baseline, and prescribed use of psychotropic medication. In this model, the AUDIT, BDI, GAF, and PSSS-R scores were treated as continuous variables. The model was adjusted for age and sex. *P* values <.05 were considered statistically significant. Statistical analyses were carried out using SPSS 14.0 software package [51] and PASW 18.0 [52].

## 3. Results

### 3.1. Characteristics at Baseline

The adolescents with and without alcohol misuse did not differ significantly in terms of gender, age, or parental socioeconomic status. Those with alcohol misuse had perceived less social support from their families (11.6 versus 13.8, *P* < .01), had worse psychosocial functioning as measured by GAF score (GAF score < 60, 87.1% versus 74.4%, *P* < .05), higher mean BDI scores (23.8 versus 21, *P* = .03), and their parents had more often problems with alcohol use (53.0% versus 36.9%, *P* < .05), (Table 1). The mean of the AUDIT score for the alcohol misusers was 14.4 compared to 2.7 of nonmisusers. Altogether, six subjects had other than alcohol related-substance use at diagnosed level, two in the alcohol nonmisusers group (both amphetamine-related disorder NOS), and four in the alcohol misusers group (three cannabis abuse, and one amphetamine-related disorder NOS).

### 3.2. Treatment Characteristics during the Follow-Up Period

By the one-year follow-up time point, those with alcohol misuse had lower treatment intensity scores (mean 1.5 versus 1.9, *P* = .04) and a poorer attendance measure (68.8% versus 76.5%, *P* < .01) than the nonmisusers. They had also more often received psychotropic medication (68.6% versus 48.8%, *P* = .01; Table 2).

### 3.3. Diagnostic and Clinical Characteristics at One-YearFollowup

Those with alcohol misuse had higher BDI score (mean 9.7 versus 7.2, *P* < .05) and higher AUDIT score (mean 10.8 versus 4.2, *P* < .001), and 55.1% of them had GAF score below 60 (versus 37.2%, *P* = .03) compared with the nonmisusers (Table 3).

### 3.4. The Logistic Regression Model for Treatment Attendance

 When age and sex were adjusted, and selected depression severity and comorbidity variables entered into the model, the strongest clinical- or treatment-related factor significantly associating with attendance (proportion of kept to scheduled individual appointments) was the baseline AUDIT score (*Wald χ* ^2^ = 56.009,   *df* = 1, *P* < .001, OR 0.97; 95% CI 0.96, 0.97; Table 4).

## 4. Discussion 

The comparisons between depressed adolescent outpatients with and without alcohol misuse yielded some important significant differences. Specifically, those with alcohol misuse had perceived less social support from their families. In addition, their parents often had more problems with alcohol use. These results are in line with previous findings indicating a number of family-related factors, such as parental substance use or abuse, poor parent-child relationships, low perceived parental support, poor communication, and poor parent supervision and management of the adolescent's behaviour, as risk factors for the development of substance abuse among adolescents in general [53].

Further, those with alcohol misuse had more depressive symptoms and poorer psychosocial functioning both at the baseline and still after a one-year follow-up period time, which is in line with the findings by Goldstein et al. [11]. Our results were expected as Meririnne et al. [12] recently reported from the ADS study that alcohol use negatively affects the course of adolescent depression and psychosocial functioning. It is also important to notice, that even after a one-year follow-up period the AUDIT scores were still very high for the alcohol misuse group, and indicated an increasing trend for the nonmisusers. Further, our study supports the previous findings that not only boys but also depressed girls appear to be at a risk for substance use involvement during adolescence [5]. This is particularly interesting, as recent observations of increasing drinking among girls have raised public concern. 

The differences in treatment received between the two groups were, as a whole, only minor: those with alcohol misuse had more often received psychotrophic medication altogether compared to the nonmisusers. These minor differences in treatment may reflect the fact that substance-related problems were either not detected at all or not taken into account when planning the treatment. This may lead to insufficient treatment, as Couwenbergh et al. [27] have pointed out. On the other hand, as the outpatients with alcohol misuse had more severe symptoms of depression, they were probably more often prescribed psychotropic medication. The possible problems recognizing alcohol use and not taken into account when formulating treatment for depressed adolescents may at least reflect (1) the lack of sufficient knowledge and expertise to assess and treat substance use problems, (2) the way the clinicians conceptualize their work (treat mental disorders versus SUDs), (3) the way the clinicians interpret the level of adolescents substance use (part of normative development versus disturbed development), (4) level of trust and therapeutic alliance between the adolescent and clinician, and (5) adolescent's own thoughts or feelings about his/her substance use leading to underreported or nondisclosure of substance use. 

Compared to, for example, practice parameters by the American Academy of Child and Adolescent Psychiatry (2007) [23], one may see at least one significant shortcoming in treatment: the exiguity of family and school involvement in treatment. According to the practise parameter for the assessment and treatment of children and adolescents with substance use disorders [53], family therapy or significant family/parental involvement are critical to the success of any treatment approach for adolescents with SUDs. Our findings on significant parental problems with alcohol among adolescents with alcohol misuse may indicate that the parents themselves were so severely distressed that the adolescents were left too alone in the family in their efforts to get help for their problems. In addition, from the adolescent developmental perspective, growing efforts to independence from the parents are commonly seen as an age-related behaviour, and the parents may have misinterpreted adolescents' alcohol use as such as a “normal” behavior. Although some degree of risk-taking may be normal in adolescence, repeated engagement in high-risk activities, persistent disregard for attempts at limit setting by authority figures, and aggressive behavior may be signs of a more serious problem [54]. Thus, both the parents and the clinicians should not interpret too easily adolescent's alcohol use as normative experimentation. 

 Interestingly, findings from this naturalistic clinical one-year follow-up study of depressed psychiatric outpatient adolescents indicate that an adolescent's severity of alcohol use, even at subdiagnostic level, has an independent negative effect on attendance at individual treatment appointments, which, for its part, most likely has a negative effect on outcome. Haw et al. [55] have concluded that both comorbidity with alcohol abuse and poor compliance with treatment may be important factors complicating therapy in many depressed patients with deliberate self-harm. As outpatient nonattendance is a serious problem in clinical and economic terms, we must at least try to speculate the reasons for poorer attendance. A number of family factors, such as parental involvement in treatment and family cohesion, have been identified as factors relating to treatment compliance among adolescent suicide attempters [56]. As mentioned above, this could be the case also in this study as depressive adolescents with alcohol misuse had perceived less social support from their families and their parents often had more problems with alcohol use. The adolescents may miss their treatment appointments also because of the direct negative impact of alcohol use on functioning (intoxication, hangover, or guilt), and the absence of subjective need for help (not seeing current pattern of alcohol use as a problem). In addition, regular alcohol use may complicate or even hinder recovery, leading to subjective disappointments in treatment.

Interestingly, in the logistic model, an association was noted between an axis II comorbidity and treatment attendance. This may suggest that special emphasis was placed on the treatment of those depressed individuals with comorbid personality disorders. This finding is to be studied in more detail in the near future.

In developing adolescent treatment settings and services, more specific treatment options are needed regarding alcohol use problems, for instance. This would require identification of different clinical presentations of disorders and subthreshold disorders, and subsequent education of personnel accordingly. As an example, failures to keep appointments should alert for potential alcohol or other substance use problem.

### 4.1. Study Limitations

The present one-year follow-up study included consequently referred depressed adolescent outpatients, whose psychiatric diagnoses and clinical characteristics were comprehensively assessed using a reliable interview instrument and self-report scales. Alcohol use was assessed by a self-report scale of AUDIT using a cutoff point of 8 points [35, 36]. Lower cutoff points have also been suggested for use among adolescents [38]; however, we aimed at studying alcohol use “severe enough” but not necessarily meeting diagnostic criteria for actual substance use disorders. It is critically important to assess the predictive meaning of alcohol misuse among depressed adolescents in order to find out effective treatments in ordinary clinical practice.

There is a possibility that the study findings are not exclusive to alcohol misuse, being perhaps influenced by other substances. However, this is unlikely as the number of other substance-related diagnoses was small.

Generalization of the finding to other cultures should be made with the understanding of possible differences between health care systems. Due to the Finnish system, adolescents believed to have a predominant and severe substance use disorders were treated elsewhere in specialized units.

This is a naturalistic clinical follow-up study, so any conclusions regarding the actual effect of the treatment on the outcome are precluded. On the other hand, naturalistic follow-up data are useful in assessing other questions, for example, factors associating with treatment adherence among “real-life” outpatients.

Due to the descriptive nature of the data, we reported the values for all the collected treatment-related variables. As these were numerous, a possibility for significant findings by chance arises. In the context of a relatively small study population, we wanted to avoid missing possible significant findings (a type II error) and decided to use basic significance tests without adjustments for the significance level, while recognizing that in so doing some of the statistically significant effects may be spurious.

## 5. Conclusions

Alcohol misuse was related to lower perceived parental support and greater parental alcohol use problems among depressed adolescent outpatients. Further, those with alcohol misuse had more depressive symptoms and poorer psychosocial functioning both at the baseline and still after one-year-follow-up period time. An adolescent's severity of alcohol use, even at subdiagnostic level as measured by self-report questionnaire and had an independent negative effect on attendance at individual treatment appointments, which, for its part, most likely had a negative effect on outcome. Early recognition of alcohol misuse is most important among adolescents in primary level treatment and other service settings. It should not be overlooked either in specialized settings focusing on the assessment and treatment of predominantly psychiatric disorders like depressive disorders. This view emphasizes the need for integrated services, having potential for simultaneous interventions and tolerance for both substance-related and other psychiatric problems. Among adolescents, the effect of family to attrition from treatment may be greater than thought. This should perhaps be taken into account by favouring family-related psychosocial methods, when possible, particularly in substance-related psychiatric problems. It seems that for depressed adolescents with alcohol and other substance-related problems specific multimodal treatment programs, accepted by adolescents and their parents, are needed.

## Figures and Tables

**Table 1:** Sociodemographic, diagnostic, and clinical characteristics of depressed outpatients by alcohol use at baseline (*n* = 156).

Characteristic	Nonmisusers (Audit <8) (86)	Alcohol misusers (Audit ≥8) (70)	*P* value
Sex, no. (%)			.51
Male	17 (19.8)	11 (15.7)	
Female	69 (80.2)	59 (84.3)	
Age at baseline, mean (SD; median)	16.3 (1.5; 17.0)	16.5 (1.7; 17.0)	.64
SES of parents, no. (%)			.19
Upper middle cl	23 (26.7)	18 (25.7)	
Lower middle cl	38 (44.2)	21 (30.0)	
Working class	19 (22.1)	25 (35.7)	
Other	6 (7.0)	6 (8.6)	
Divorce of parents, no. (%)	36 (41.9)	31 (44.3)	.65
Parents' mental health problems, no. (%)	35 (42.2)	28 (43.1)	.91
Parents' alcohol use problems, no. (%)	31 (36.9)	35 (53.0)	**.048**
Perceived social support			
Total, mean (SD; median)	44.7 (11.2; 46.5)	43.5 (8.9;44.5)	.17
Close/intimate friend	16.1 (4.9; 18.0)	16.7 (4.1;18.0)	.63
Family	13.8 (4.4; 14.0)	11.6 (4.7;11.5)	**.004**
Friends	14.9 (5.1; 16.5)	15.2 (4.8;16.0)	.87
Age of 1st mood disorder, mean (SD; median)	13.4 (2.5; 13.5)	13.2 (2.9; 13.0)	.67
Depression diagnosis, no. (%)			.75
MDD single	45 (52.3)	36 (51.4)	
MDD recurrent	21 (24.4)	20 (28.6)	
Dysthymia/Douple dep	13 (15.1)	7 (10.0)	
Minor	7 (8.1)	7 (10.0)	
Axis I: any comorbidity, no. (%)	62 (72.1)	52 (74.3)	.76
Any SUD, no. (%)	4 (4.7)	22 (31.4)	<.001
Axis II: any comorbidity, no. (%)	30 (34.9)	28 (41.2)	.42
Axis III: medical comorbidity, no (%)	29 (33.7)	14 (20.0)	.56
Axis V: GAF score <60, no. (%)	64 (74.4)	61 (87.1)	**.048**
BDI score at baseline, mean (SD; median)	21.0 (8.7; 19.5)	23.8 (8.8; 24.5)	**.03**
AUDIT score at baseline, mean (SD; median)	2.7 (2.4; 2.0)	14.4 (5.9; 12.0)	**<.001**
BAI score at baseline, mean (SD; median)	20.8 (11.4; 19.0)	22.9 (12.2; 20.0)	.38
SSI score at baseline, mean (SD; median)	4.1 (7.1; 0)	5.8 (7.2; 3.0)	.12

**Table 2:** Treatment received of depressed outpatients by alcohol use during the one-year follow-up period (*n* = 156).

Characteristic	Nonmisusers (Audit < 8) (86)	Alcohol misusers (Audit ≥ 8) (70)	*P* value
*Psychosocial treatment *			
(i) Kept individual appointments, no. (%)			.35
<10	15 (17.4)	17 (24.3)	
10–25	42 (48.8)	36 (51.4)	
>25	29 (33.7)	17 (24.3)	
(ii) Kept family/network appoint, mean (SD; median)	1.4 (1.9; 1.0)	1.2 (2.1;0.0)	.34
(iii) Intensity (individual appoint/month), mean (SD; median)	1.9 (1.1; 1.8)	1.5 (0.8; 1.3)	**.04**
(iv) Attendance % (individual appoint), mean (SD; median)	76.5 (15.6; 76.0)	68.8 (15.8; 68.5)	**.004**
*Psychotropic medications*			
Any, no (%)	42 (48.8)	48 (68.6)	**.01**
(i) Antidepressants	39 (45.3)	41 (58.6)	.10
SSRI	38 (44.2)	39 (55.7)	.15
other antidepressants	8 (9.3)	13 (18.6)	.17
(ii) anxiolytics/ sedatives	20 (23.3)	26 (37.1)	.06
(iii) antipsychotics	8 (9.3)	11 (15.7)	.22
(iv) other	4 (4.7)	3 (4.3)	.66
*Combined treatment, no. (%)*			.21
Individual psychotherapy	20 (23.3)	17 (24.3)	
+ family counselling	27 (31.4)	12 (17.1)	
+ medication	19 (22.1)	21 (30.0)	
+ medic + family	20 (23.3)	20 (28.6)	
*Hospitalization, no. (%)*	13 (15.1)	10 (14.3)	.88
*Number of hospitalization days, *mean (SD; median)	13.3 (48.5; 0.0)	10.4 (32.9; 0.0)	.89
*Treatment status at the one-year followup, *no. (%)			.47
(i) No treatment	30 (34.9)	24 (34.3)	
(ii) Psychiatric outpatient	55 (64.0)	43 (61.4)	
(iii) Psychiatric inpatient	1 (1.2)	3 (4.3)	

**Table 3:** Diagnostic and clinical characteristics of depressed outpatients at the one-year followup (by the alcohol use at baseline) *n* = 156.

Characteristic	Nonmisusers (Audit < 8) (86)	Alcohol misusers (Audit ≥ 8) (70)	*P* value
BDI score, mean (SD; median)	7.2 (7.8; 6.0)	9.7 (9.5; 7.0)	**.045**
Axis V: GAF score < 60, no. (%)	32 (37.2)	38 (55.1)	**.03**
AUDIT score, mean (SD; median)	4.2 (4.5; 3.0)	10.8 (6.4; 10.0)	<.001
Diagnostic status, no. (%)			.73
recovery	32 (37.2)	26 (37.1)	
persistent depression	42 (48.8)	37 (52.9)	
recurrence	12 (14.0)	7 (10.0)	
Any SUD, no. (%)	1 (1.2)	13 (18.6)	<.001

**Table 4:** Logistic regression analysis of treatment attendance^1^ during one-year followup on baseline clinical and treatment-related characteristics.

	*Waldχ* ^2^	*P*	*OR* [*95*% *CI*]
Age at baseline	0.960	.327	1.03 [0.98–1.08]
Sex	0.626	.429	0.92 [0.74–1.14]
BDI score	0.245	.621	1.00 [1.00–1.01]
AUDIT score	56.009	<.001	0.97 [0.96–0.97]
Axis I: any comorbidity	2.251	.134	1.14 [0.96–1.36]
Axis II: any comorbidity	17.143	<.001	1.44 [1.21–1.71]
Axis V: GAF score	2.259	.133	0.13 [1.0–1.02]
Perceived social support from the family	0.142	.706	1.00 [0.99–1.02]
Psychotropic medication, any	0.017	.895	0.99 [0.83–1.18]

^1^Proportion of kept to scheduled individual appointments.
